# The effects of sarcopenia on the prognosis of patients with acute-on-chronic liver failure: a systematic review and meta-analysis

**DOI:** 10.3389/fnut.2025.1649783

**Published:** 2025-10-14

**Authors:** Wenyan Li, Rong Deng, Yunting Luo, Hong Li, Fang Chen, Jing Zhao, Min Liu, Qianxi Liu, Yaojie Mao, Lixia Zhang

**Affiliations:** ^1^Center of Infectious Diseases, West China Hospital/West China School of Nursing, Sichuan University, Chengdu, China; ^2^Center of Infectious Diseases, West China Hospital, Sichuan University, Chengdu, China

**Keywords:** ACLF, mortality, meta-analysis, prognosis, sarcopenia, systematic review

## Abstract

**Objectives:**

Sarcopenia is an important indicator affecting the prognosis of patients with end-stage liver disease. The purpose of this review is to systematically review all relevant studies to evaluate the impact of sarcopenia on the prognosis of patients with ACLF.

**Methods:**

We systematically searched PubMed, Web of Science, Embase, the Cochrane Library, China Biomedical Literature Service System (CINAHL), China National Knowledge Infrastructure (CNKI), the WeiPu (VIP) and the Wanfang database for relevant articles published on or before August 1, 2024. Two independent reviewers conducted literature screening against predefined inclusion/exclusion criteria.

**Results:**

The systematic review included 12 cohort studies, with a total of 2,505 participants. Of these, 11 studies (2,072 participants) met the criteria for inclusion in the meta-analysis. The meta-analysis demonstrated that sarcopenia is significantly associated with an increased mortality risk in patients with ACLF [HR = 1.27, 95%CI (1.05, 1.54), *p* < 0.00001]. Subgroup analyses showed that the mortality risk was significantly increased in the subgroup of study with the outcome of short-term mortality [HR = 1.54, 95%CI (1.14, 2.08)], and prospective study [HR = 2.16, 95%CI (1.30, 3.60)].

**Conclusion:**

The meta-analysis of 11 studies (2,072 participants) revealed a significant association between sarcopenia and elevated mortality risk in ACLF patients. However, these findings should be interpreted with caution due to the limited number of included studies and higher heterogeneity in the analysis.

**Systematic review registration:**

CRD42023441039.

## Introduction

In 2010, the European Working Group on Sarcopenia in Older People (EWGSOP) defined sarcopenia (1), and the definition was updated by EWGSOP in 2019 (2). Since then, sarcopenia is considered a progressive and generalized skeletal muscle disorder characterized by the gradual loss of muscle strength, mass and function (2). Sarcopenia is formally recognized as a muscle disease with an ICD-10-MC Diagnosis Code (3). It is associated with an increased likelihood of adverse outcomes including falls (4), fractures (5), mobility disorders (6), physical disability (7), and many other adverse outcomes, even mortality (8). As we all know, sarcopenia poses a significant threat to the health and independence of the elderly.

In recent years, sarcopenia in patients with chronic liver disease has received more and more attention. Some studies found that patients with cirrhosis frequently present with sarcopenia, the prevalence of sarcopenia is 40–70% (9). Also, sarcopenia is an important indicator affecting the prognosis of patients with end-stage liver disease (ESLD) (10). Sarcopenia can predict disease progression, complications of cirrhosis [such as the incidence of hepatic encephalopathy (HE)], mortality of cirrhotic patients, long-term outcomes after liver transplantation, and outcomes of patients with hepatocellular carcinoma (HCC) (11–14). Although the prognostic value of sarcopenia in terms of ESLD is known, its impact on the development of acute-on-chronic liver failure (ACLF) is still limited.

Acute-on-chronic liver failure (ACLF) (15) is a clinical syndrome characterized by acute hepatic decompensation in chronic liver disease, with or without cirrhosis and mainly manifests as coagulopathy, jaundice, ascites, and HE. ACLF has the characteristics of rapid disease progression and a high case fatality rate. Therefore, active exploration of indicators determining the prognosis of the patients with ACLF is valuable to guide treatment. Studies have suggested that sarcopenia is significantly associated with both short-term (28 days [HR = 2.05, 95%CI (1.41–3.00), *p* < 0.01], 90 days [HR = 1.802, 95% CI (1.062–3.060), *p* = 0.029]) and long-term (1 year [HR = 1.81, 95% CI (1.29–2.54), *p* < 0.01] and overall [HR = 1.82, 95% CI (1.30–2.55), *p* < 0.01]) (16, 17) mortality rates in patients with ACLF. Nevertheless, the study of Xu et al. (18) did not find an independent relationship between psoas muscle index (PMI) and 1-year mortality. Current research on the association between sarcopenia and the prognosis of patients with ACLF is controversial.

As far as we can determine, there is a paucity of comprehensive data to explore systematically the impact of sarcopenia on the prognosis of patients with ACLF. Given this background, the main aim of this systematic review is to describe systematically the effects of sarcopenia on the prognosis of patients with ACLF.

## Methods

### Conduct of the systematic literature review

The systematic review was conducted according to the Preferred Reporting Items for Systematic Review and Meta-Analysis (PRISMA) guidelines (19). The protocol was registered in the International Prospective Register of Systematic Reviews (PROSPERO) (registration number: CRD42023441039).

### Definition of sarcopenia

According to the definition of the European Working Group of Sarcopenia in Older People (EWGSOP2) (2), sarcopenia is considered a progressive and generalized skeletal muscle disorder characterized by the gradual loss of muscle strength, mass and function.

Based on previous studies, we included studies that reported sarcopenia of any severity under any definition criteria.

### PICOS question and eligibility criteria

Following the PICOS question, we included:

- Participants: patients ≥18 years of age who were diagnosed with ACLF.
- Intervention: presence of sarcopenia using any definition.
- Controls: patients without sarcopenia.
- Outcomes: mortality was the primary outcome, and infection was the secondary outcome. The effect size was presented as the hazard ratio (HR) and 95% confidence interval (CI).
- Study design: a prospective or retrospective cohort study.

We excluded the following studies: conference papers, case reports, letters to the Editor, reviews, studies that did not provide sufficient data, non-Chinese and non-English literature.

### Data sources and search strategy

Several relevant databases were comprehensively searched for this systematic review, including PubMed, Web of Science, Embase, the Cochrane Library, the China Biomedical Literature Service System, China Biomedical Literature Service System (CINAHL), China National Knowledge Infrastructure (CNKI), the WeiPu (VIP) and the Wanfang database, from inception to 1 August 2024.

The following search was used in PubMed:

((((liver failure[Title/Abstract]) OR (hepatic failure[Title/Abstract])) OR (acute-on-chronic liver failure[Title/Abstract])) OR (ACLF[Title/Abstract])) OR (end-stage liver disease[Title/Abstract]) OR ((((liver failure[MeSH Terms]) OR (hepatic failure[MeSH Terms])) OR (acute-on-chronic liver failure[MeSH Terms])) OR (end-stage liver disease[MeSH Terms]))

AND

(((((((((((sarcopenia[Title/Abstract]) OR (sarcopenic[Title/Abstract])) OR (muscle loss[Title/Abstract])) OR (muscular atrophy[Title/Abstract])) OR (less muscle disease[Title/Abstract])) OR (muscle wasting[Title/Abstract])) OR (muscle mass[Title/Abstract])) OR (muscle weakness[Title/Abstract])) OR (muscle strength[Title/Abstract])) OR (hand strength[Title/Abstract])) OR (grip strength[Title/Abstract]) OR ((((((((sarcopenia[MeSH Terms]) OR (muscular atrophy[MeSH Terms])) OR (less muscle disease[MeSH Terms])) OR (muscle wasting[MeSH Terms])) OR (muscle mass[MeSH Terms])) OR (muscle weakness[MeSH Terms])) OR (muscle strength[MeSH Terms])) OR (hand strength[MeSH Terms])) OR (grip strength[MeSH Terms])).

### Study selection

Two researchers screened the articles and extracted data independently, utilizing EndNote software. Disagreements between researchers were resolved through discussion or consultation with a third reviewer in necessity. Data is extracted from eligible articles using a standardized form. The form included: the first author, publication year, types of study, age, regions, observation period, sample size, percentage of women, definitions and measurements of sarcopenia, ACLF definition, outcomes, follow-up period, reported HR (95%CI), and adjusted variables.

### Methodological quality assessment

Two researchers independently evaluated the quality of cohort studies by the Newcastle-Ottawa Scale (NOS) (20). A discussion was made if there was a disagreement. The scale covers three domains and eight items, including the selection of research subjects (4 items, 4 points), comparability between groups (1 item, 2 points), and outcome measurement (3 items, 3 points). The total score for every study was 9. The study was considered moderate to high quality if the score was more than six.

### Statistical analysis

The data analysis was conducted using RevMan 5.4 and Stata 12.0. Meta-analysis was performed using a random-effects model to generate a pooled hazard ratio (HR) and 95% confidence interval (CI). When I^2^ ≥ 50% or *p* < 0.05, heterogeneity was determined to be statistically significant. To explore the source of heterogeneity in the study, we performed a series of subgroup analyses. In addition, to evaluate publication bias by observing the symmetry of the funnel plot, Begg’s/ Egger’s tests and combined with trim and fill method. Sensitivity analysis was performed by excluding each study to evaluate the stability of the results. For all statistical tests, the test level of meta-analysis was set at *α* = 0.05.

## Results

### Description of studies and study population

**Figure 1:**
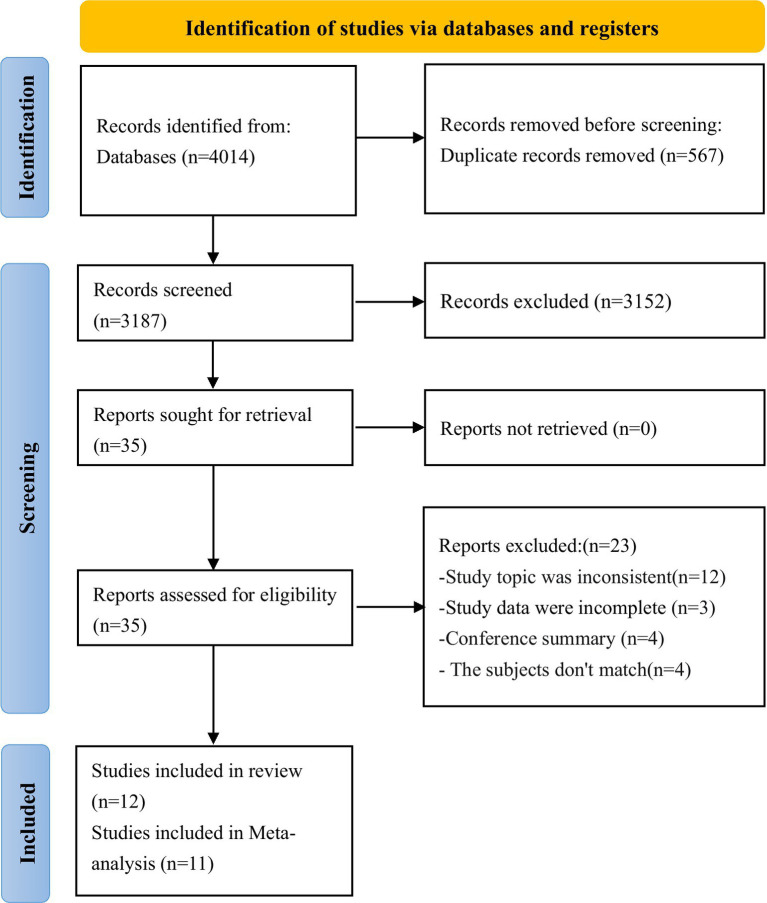
Flowchart of study selection.

Figure 1 summarizes the yield of the search process. A total of 4,014 records were identified from the database, including 567 duplicates. Following the removal of duplicates, 3,187 records passed through title and abstract screening, and 35 articles were retained for full-text review. Ultimately, 12 total studies were included in the qualitative and quantitative syntheses, collectively representing approximately 2,505 participants. Of these, 11 studies were identified for meta-analysis and included approximately 2,072 participants. The study of Peng et al. (21) was excluded from the meta-analysis, as its outcomes significantly differed from those reported by other 11 studies.

Table 1 reports the main characteristics of the included studies in the systematic review.

**Table 1:** Characteristics of studies included in the systematic review (*N* = 12).

First author (year)	Study design	Region	Observation period	Sample size	Female/%	Age	Measurements of sarcopenia	ACLF definition	Outcomes	Follow-up period
Artru, F. (2024) (22)	Retrospective study	France	2008.01–2015.12	73	30.1	56.8 (48.6,62.0)	PMI (CT)	EF-CLIF	10-year mortality	7.5 years (2.2–10.0)
Geng, N. (2023) (23)	Prospective study	China	2021.03–2022.10	126	——	46.1 ± 10.9	SMI (CT)	APASL	90 days-mortality	90 days
Geng, N. (2024) (17)	Retrospective study	China	2019.01–2022.01	431	16.0	47.0 ± 10.0	L3-SMI (CT)	APASL	90 days-mortality	90 days
Li, T. Z. (2021) (24)	Retrospective study	China	2015.01–2019.06	171	14.6	44.5 ± 10.8	L3-SMI (CT)	APASL	Mortality	3 months
Liu, L. (2022) (25)	Retrospective study	China	2013.01–2019.12	271	17.7	51.8 ± 10.7	L3-SMI (CT)	APASL	90 days-mortality	90 days
Praktiknjo, M. (2018) (26)	Retrospective study	Germany	1993.12–2014.09	116	40.5	59.0 (18.0,79.0)	FFMA/MA (MRI/CT)	——	3 year-mortality	3 years
Wang, J. S. (2022) (27)	Prospective study	China	2021.01–2021.06	110	23.6	50.0 ± 12.4	L3-SMI (CT/MRI)	CMA	90 days-mortality	90 days
Wang, J. (2024) (28)	Retrospective study	China	2017.12–2021.12	126	——	50.0 ± 11.0	L3-SMI (CT)	APASL	Mortality, Infection	4 years
Xu, M. M. (2022) (18)	Retrospective study	China	2015.01–2019.06	116	0.0	43.6 ± 10.4	PMI (CT)	APASL	360 days-mortality	360 days
Zeng, F. (2024) (16)	Retrospective study	China	2016.01–2022.09	414	23.2	52.9 ± 13.4	L3-SMI (CT)	APASL	28 days-mortality, 1 year-mortality, overall mortality	6 years
Zhou, C. (2023) (29)	Prospective study	China	2012.11–2014.12	118	11.9	42.9 ± 10.7	ESI (CT)	APASL	90 days-mortality, kidney dysfunction, hepatic encephalopathy, hospital infection	90 days
Peng, H. (2022) (21)	Retrospective study	China	2019.07–2021.03	433	20.1	47.0 (34.8,54.3)	L3-SMI (CT)	APASL	90 days-progression (LT/death)	90 days

PMI, Psoas muscle index; SMI, Skeletal muscle index; EF-CLIF, European Foundation for Research in Chronic Liver Failure; APASL, Asia-pacific Association for the Study of Liver; L3-SMI, Lumbar 3-skeletal muscle index; FFMA, Fat-free muscle area; MA, Muscle area; CMA, Chinese Medical Association; ESI, Transversal erector spine index; LT, Liver transplantation; CT, Computerized tomography; MRI, Magnetic resonance imaging.

### Study quality assessment

The Newcastle–Ottawa Scale (20) was used to assess the risk of bias for the 12 studies included in the systematic analysis, with the results presented in Table 2. Two studies scored 8 or 9 points, indicating a low risk of bias. Ten studies scored 6 or 7 points, indicating a medium risk of bias. Consequently, since all cohort studies achieved a score of 6 or higher, no studies were excluded from the systematic review.

**Table 2:** Methodological quality score of the included studies based on the Newcastle–Ottawa Scale (NOS) tool.

Author (year)	Selection				Comparability	Exposure/outcome			
	Representativeness of cohort	Selectin of control cohort	Ascertainment of exposure	Outcome not present at start	Comparability of cohorts	Assessment of outcome	Length of follow up	Adequacy of follow up	Total score
Artru, F. (2024) (22)	1	1	1	1	0	1	1	0	6
Geng, N. (2023) (23)	1	1	1	1	2	1	0	0	7
Geng, N. (2024) (17)	1	1	1	1	2	1	0	0	7
Li, T. Z. (2021) (24)	1	1	1	1	2	1	0	0	7
Liu, L. (2022) (25)	1	1	1	1	0	1	0	1	6
Praktiknjo, M. (2018) (26)	1	1	1	1	2	1	0	0	7
Wang, J. S. (2022) (27)	1	1	1	1	2	1	0	1	8
Wang, J. (2024) (28)	1	1	1	1	2	1	0	0	7
Xu, M. M. (2022) (18)	1	1	1	1	2	1	0	0	7
Zeng, F. (2024) (16)	1	1	1	1	2	1	1	1	9
Zhou, C. (2023) (29)	1	1	1	1	2	1	0	0	7
Peng, H. (2022) (21)	1	1	1	1	2	1	0	0	7

### Meta-analysis

#### Association between sarcopenia and mortality risk of patients with ACLF

Twelve studies (16–18, 21–29) reported the association between sarcopenia and prognosis of patients with ACLF. Among them, 11 studies (16–18, 22–29) reported the association between sarcopenia and mortality risk of patients with ACLF. Nevertheless, the outcome of the study of Peng et al. (21) was ACLF progression, including death and liver transplantation. Therefore, the study by Peng et al. (21) was excluded from the meta-analysis examining the association between sarcopenia and mortality risk in patients with ACLF. The meta-analysis demonstrated that sarcopenia is significantly associated with an increased mortality risk in patients with ACLF [HR = 1.27, 95%CI (1.05, 1.54), *p* < 0.00001].

**Figure 2:**
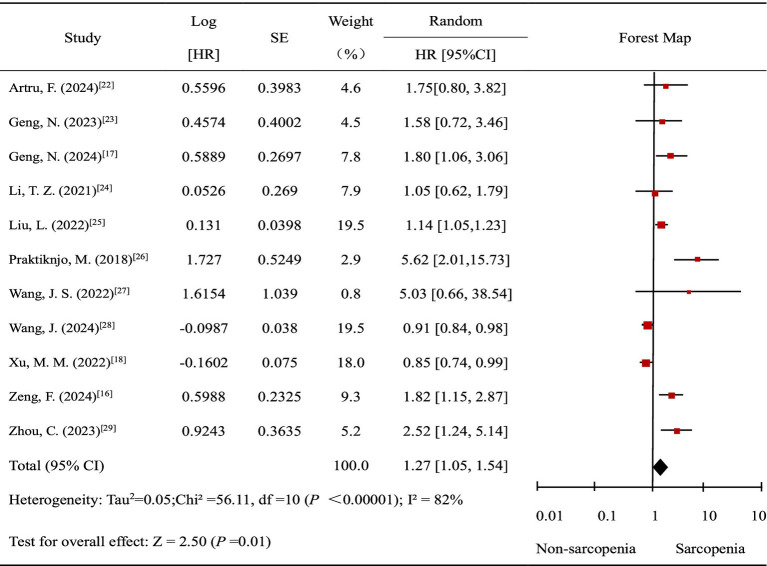
Meta-analysis of the effects of sarcopenia on mortality in patients with ACLF.

Overall, in comparison to the non-sarcopenic group, the sarcopenic is significantly associated with an increased mortality risk in patients with ACLF [HR = 1.27, 95%CI (1.05, 1.54), *p* < 0.00001], and the heterogeneity is high (I^2^ = 82%) (Figure 2).

### Subgroup analysis

Subgroup analyses showed that the mortality risk was significantly increased in the study subgroup with short-term mortality outcomes [HR = 1.54, 95%CI (1.14, 2.08)] and prospective study [HR = 2.16, 95%CI (1.30, 3.60)]. Other subgroup analyses did not show a significant association between sarcopenia and the mortality risk.

By conducting subgroup analyses stratified by outcomes, regions, methods for measuring sarcopenia, and sample size, we did not find a significant reduction in heterogeneity, indicating that heterogeneity was not associated with these variables. Heterogeneity was significantly reduced in subgroup analyses of research type, suggesting that heterogeneity might have originated from research type. This may be related to the potential recall bias inherent in retrospective cohort studies compared to prospective cohort studies. Furthermore, the high heterogeneity may also be related to variations in the original studies, including non-standardized definitions of sarcopenia, differences in baseline disease severity among patients, and other potential comorbidities (malnutrition/ inflammation) (Table 3).

**Table 3:** Subgroup analyses of the association between sarcopenia and mortality risk of patients with ACLF: Random-effects model.

	Variables	HR	95%CI	I^2^ (%)	No. studies
Outcomes	Short-term mortality (≤90d-mortality)	1.54	1.14, 2.08	58	7
	Long-term mortality (>90d-mortality)	1.21	0.91, 1.61	84	5
Regions	China	1.17	0.98, 1.40	82	9
	Other	2.98	0.95, 9.33	68	2
Measurements of sarcopenia	SMI	1.21	0.99, 1.49	81	7
	Other	1.97	0.85, 4.59	87	4
Sample size	<200	1.24	0.96, 1.61	75	8
	≥200	1.45	1.00, 2.11	70	3
Research type	Prospective study	2.16	1.30, 3.60	0	3
	Retrospective study	1.18	0.98, 1.43	85	8

HR, hazard ratio; CI, confidence interval; SMI, Skeletal muscle index.

#### Sensitivity analysis and publication bias

Through sensitivity analysis, we found that the combined HR did not change significantly for any individual study, thus supporting the meta-analysis results.

**Figure 3:**
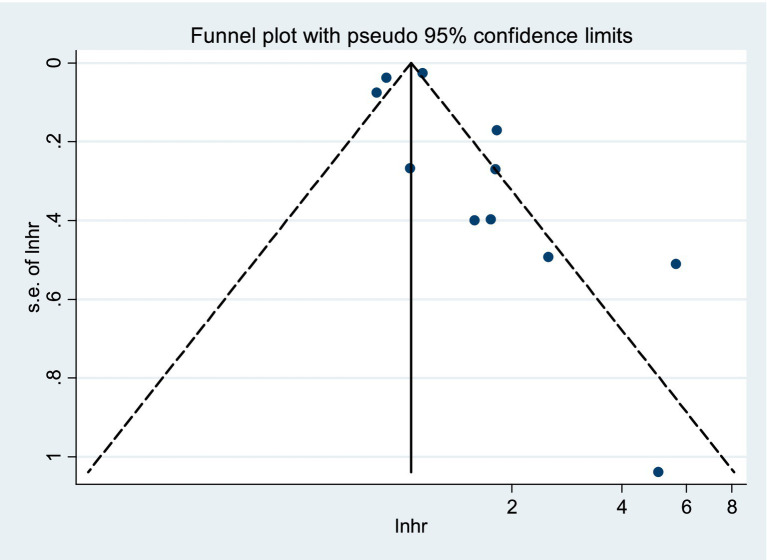
Hazard ratio (HR) of the association between sarcopenia and mortality of patients with ACLF.

Gross examination of the funnel plot revealed potential evidence of publication bias (Figure 3). Nevertheless, no significant publication bias was found by Begg’s test (*p* = 0.533 > 0.05) and Egger’s test (*p* = 0.150 > 0.05). In addition, after adjusting for publication bias using the trim-and-fill method, the originally significant positive result became non-significant [HR = 1.046, 95%CI (0.873–1.253), *p* = 0.627 > 0.05]. Consequently, the study findings may be influenced by publication bias, and additional high-quality, large-scale investigations are warranted to validate the effect size.

### Descriptive analysis of the association between sarcopenia and infection of patients with ACLF

In the 12 included studies, only 2 cohort studies (28, 29) reported infection-related outcomes.

Of these, the research of Wang et al. (28) suggested that low L3-SMI is an independent risk factor for the development of infections and significantly influences [OR = 0.89, 95%CI (0.81–0.97), *p* = 0.011]. However, Zhou, et al. (29) found no significant difference in the incidence of in-hospital infections between patients with low ESI and those with ACLF [HR = 1.62, 95%CI (0.6–3.08), *p* = 0.138]. Therefore, the association between sarcopenia and infection in patients with ACLF remains unclear. Further research is necessary to elucidate this association.

## Discussion

### Principal findings

The systematic review and meta-analysis reviewed data from 12 cohort studies and analyzed the effect of sarcopenia on the prognosis of patients with ACLF. Our meta-analysis found that sarcopenia is significantly associated with an increased mortality risk in patients with ACLF. However, we did not find a significant association between sarcopenia and the risks of infection in patients with ACLF.

### Potential mechanisms

In recent years, an increasing number of studies have been conducted to explore the association between sarcopenia and other diseases, such as chronic liver disease (30), neoplastic diseases (31), and liver transplantation (32), et al. Sarcopenia is closely associated with the prognosis of these diseases. However, few studies have specifically examined the effect of sarcopenia on patients with ACLF. Currently, the mechanisms through which sarcopenia influences the prognosis of patients with ACLF remain unclear.

The mechanisms by which sarcopenia influences the prognosis of patients with ACLF may be attributable to systemic inflammation, and hyperammonemia, et al. First, systemic inflammation is the key contributor to the loss of skeletal muscle and its functionality (33). Also, Systemic inflammation is one of the features of ACLF and is intimately associated with the prognosis of patients (34). For patients with ACLF, various risk factors can lead to acute hepatic decompensation, which in turn causes systemic inflammation based on chronic liver disease. ACLF often manifests as acute systemic inflammatory response syndrome (35). Of these, pro-inflammatory factors [tumor necrosis factor (TNF-*α*), interleukin (IL-1β), and interferon (IFN-*γ*)] (33) can inhibit the expression of growth hormone and promote the occurrence of sarcopenia. Meanwhile, pro-inflammatory factors also activate the ubiquitin-proteasome pathway, causing protein degradation, leading to sarcopenia (36). When patients with ACLF are complicated by sarcopenia, their bodies are still unable to cope with the catabolic changes, even if liver function is compensated (37). Therefore, sarcopenia may be a manifestation of potential systemic inflammation, which could influence the prognosis of patients with ACLF. Furthermore, Ye et al. (38) conducted a cohort study of 140 patients with ACLF and suggested that white blood cells (WBC) were significantly elevated in patients with sarcopenia, which indirectly confirmed the potential relationship among sarcopenia-inflammation-ACLF. Second, as a common complication of patients with ACLF, hyperammonemia may reduce muscle protein synthesis by upregulating myostatin production (39). Finally, patients with ACLF often experience a reduced oral intake due to complications of the portal system and impaired liver function (40). This diminished dietary protein intake is also among the contributing factors to the onset of sarcopenia. Also, systemic inflammation and hyperammonaemia can contribute to a heightened catabolic state and collectively lead to malnutrition and sarcopenia (16).

### Mortality

As we all know, patients with ACLF have a very high short-term mortality when treated without liver transplantation (41). Our study found that the short-term mortality risk (≤90 days mortality) [HR = 1.54, (1.14, 2.08)] is higher than the total mortality risk [HR = 1.27, 95%CI (1.05, 1.54)] of patients with ACLF who are complicated by sarcopenia. Therefore, sarcopenia may be a reliable indicator to predict the prognosis of patients with ACLF in the future. The death of patients with ACLF is associated with multi-organ failure. Sarcopenia reduces the body’s compensatory capacity, making patients more susceptible to severe complications such as hepatorenal syndrome and hepatic encephalopathy, thereby significantly increasing 28-day and 90-day mortality rates. Studies suggested that the mortality of ACLF increased with the increasing incidence of organ failure. Patients with one organ failure have a mortality rate of about 20% within 28 days, while those with three organ failures have a mortality rate of more than 70% (42). Liver transplantation has been demonstrated to improve the survival rates of patients with ACLF significantly, but the applicability and feasibility of liver transplantation are limited by various factors.

### Infection

Infection was deemed to be a major cause of increased mortality in patients with ACLF (43). Identification of infection-related risk factors in ACLF patients could facilitate the development of multidisciplinary therapeutic strategies, thereby potentially improving clinical outcomes and reducing mortality. A recent study shows that sarcopenia is a highly predictive nutritional indicator for the occurrence of hospital-acquired infections (44). The study of Wang et al. (28) suggested that sarcopenia is an independent risk factor for infections in ACLF patients. The elevated infection risk associated with sarcopenia may be mediated through immune system compromise, as muscle wasting has been shown to impair both innate and adaptive immune responses in chronic disease states (45). However, the study of Zhou et al. (29) found no significant difference in the incidence of in-hospital infections between patients with low ESI and those with ACLF. Differences across studies stems from multiple sources, including the operational definition and measurement modality used for sarcopenia, baseline demographic and clinical characteristics of the enrolled populations, sample size, and other methodological factors. Thus, the causal relationship between sarcopenia and infection—whether sarcopenia predisposes to infections or infections accelerate muscle wasting—remains to be elucidated through prospective studies. Clarifying this bidirectional association could inform novel therapeutic and preventive strategies.

### Strengths and limitations

The current meta-analysis has the following strengths. First, according to our knowledge, our research is the first meta-analysis to evaluate the effects of sarcopenia on the prognosis of patients with ACLF. Second, our study strictly adhered to the Preferred Reporting Items for Systematic Reviews and Meta-Analysis (PRISMA) 2020 guidelines. Finally, we used several methods to fully test the stability of the results, encompassing sensitivity analyses and subgroup analyses.

Besides, our research still presents some potential limitations. First, these findings should be interpreted with caution due to the limited number of included studies and higher heterogeneity in the analysis. Besides, we conducted further analysis to evaluate the source of this heterogeneity. The subgroup analysis found that interstudy heterogeneity may be associated with research type. Second, we cannot extract the HR from the study about sarcopenia and infection in ACLF patients. Therefore, we only conducted a descriptive analysis to explore the association between sarcopenia and infection in ACLF patients. Third, there may be potential publication bias in our meta-analysis. Finally, the majority of the studies we included were retrospective cohort studies, which means we cannot infer the causal relationship between sarcopenia and the mortality of patients with ACLF. We recommend that future high-quality prospective studies be conducted to validate our findings.

## Conclusion

Our meta-analysis showed that sarcopenia is significantly associated with the mortality risk of patients with ACLF. Sarcopenia may increase the risk of mortality in patients with ACLF. Nevertheless, sarcopenia screening is not currently recommended for inclusion in prognostic models for ACLF. And, we must carefully interpret the result because the number of studies included is small. The study’s findings underscore the importance of recognizing sarcopenia among patients with ACLF, thereby heightening clinicians’ awareness of its prognostic implications and guiding interventions to improve outcomes.
